# Study of Coating Growth Direction of 6061 Aluminum Alloy in Soft Spark Discharge of Plasma Electrolytic Oxidation

**DOI:** 10.3390/ma17122947

**Published:** 2024-06-16

**Authors:** Wenqiang Wang, Yifeng Yang, Cancan Liu, Bo Chen, Xuanyu Chen, Hao Wang, Rui Tong, Shiquan Zhou

**Affiliations:** College of Materials Science and Engineering, Nanjing Tech University, Nanjing 211800, China; 202161203202@njtech.edu.cn (W.W.); 202261103119@njtech.edu.cn (Y.Y.); 202161203196@njtech.edu.cn (B.C.); 202161203207@njtech.edu.cn (X.C.); 202261203312@njtech.edu.cn (H.W.); 202261203248@njtech.edu.cn (R.T.); 202261203164@njtech.edu.cn (S.Z.)

**Keywords:** plasma electrolytic oxidation, soft spark discharge, growth direction, α-Al_2_O_3_

## Abstract

Conventional plasma electrolytic oxidation treatments produce oxide coatings with micron-scale discharge pores, resulting in insulation and wear and corrosion resistance far below that expected of highly dense Al_2_O_3_ coatings. The introduction of cathodic polarization during the plasma electrolytic oxidation process, especially when the applied cathode-to-anode current ratio (Rpn) is greater than 1, triggers a unique plasma discharge phenomenon known as “soft sparking”. The soft spark discharge mode significantly improves the densification of the anode ceramic layer and facilitates the formation of the high-temperature α-Al_2_O_3_ phase within the coating. Although the soft spark discharge phenomenon has been known for a long time, the growth behavior of the coating under its discharge mode still needs to be studied and improved. In this paper, the growth behavior of the coating before and after soft spark discharge is investigated with the help of the micro-morphology, phase composition and element distribution of a homemade fixture. The results show that the ceramic layer grows mainly along the oxide–electrolyte direction before the soft spark discharge transformation; after the soft spark discharge, the ceramic layer grows along the oxide–substrate direction. It was also unexpectedly found that, under soft spark discharge, the silicon element only exists on the outside of the coating, which is caused by the large size and slow migration of SiO_3_^2−^, which can only enter the ceramic layer and participate in the reaction through the discharge channel generated by the strong discharge. In addition, it was also found that the relative phase content of α-Al_2_O_3_ in the coating increased from 0.487 to 0.634 after 10 min of rotary spark discharge, which is an increase of 30.2% compared with that before the soft spark discharge transition. On the other hand, the relative phase content of α-Al_2_O_3_ in the coating decreased from 0.487 to 0.313 after 20 min of transfer spark discharge, which was a 55.6% decrease compared to that before the soft spark discharge transformation.

## 1. Introduction

Plasma electrolytic oxidation (PEO), also known as micro-arc oxidation, is a development of anodizing technology. In recent years, with the introduction of negative currents, the traditional perception that the growth of oxides in PEO coatings occurs only under anodic polarization has been falsified. Troughton et al. [1] successfully demonstrated the existence of cathodic discharge in the PEO process by high-speed photography and gas emission detection. Cathodic discharge can effectively reduce the intensity of spark discharge. This mainly depends on the discharge mode brought about by the cathodic discharge. Hussein et al. [2,3] found that the generation of B-type discharge can be suppressed under the bipolar current mode, which can reduce the structural defects in coatings. Qian et al. [4] found that the negative current can increase the growth rate of PEO coatings on the one hand and reduce the discrete pores inside the coatings on the other. Tsai et al. [5], Melhem et al. [6] and Bahador et al. [7] all overcame the defects formed when the anodic charge is highger than the cathodic charge during the plasma electrolytic oxidation of aluminum alloys by increasing the cathodic charge. Interestingly, when the cathodic current is higher than the anodic current, a unique discharge phenomenon called a “soft spark” is generated during the plasma electrolytic oxidation of aluminum alloys. It has been proved by Qian et al. [4] and Song et al. [8] that the relative content of α-Al_2_O_3_ in the PEO process of aluminum alloys increases with the prolongation of the soft spark discharge time, and the mechanism of soft spark formation on the surface of aluminum alloys has been further investigated by Martin et al. [9,10,11,12] and Tjiang et al. [13]. The mechanism was investigated, and it was found that the soft sparking condition is favorable for filling the cavities of a coating and that the filled cavities can effectively reduce the porosity of the coating, thus enhancing the adhesion between the substrate and the PEO coating. This has been well established in the studies of Song et al. [8] and Qian et al. [4]. In addition, Cheng et al. [14] showed that the abrasion resistance of PEO coatings is related to the holes in the coatings. And the filling of cavities also favors the wear resistance of coatings, which was also confirmed in the study of Qian et al. [4]. Due to the excellent performance of an aluminum alloy after soft spark treatment with PEO, a large number of scholars have been attracted to studying the soft spark phenomenon.

Aluminum alloy soft spark characteristics are usually manifested as follows [15]: (a) the spark changes from a large orange spark to a uniformly distributed fine snow-white spark; (b) the acoustic emission is reduced and the photoemission is decreased; (c) the anode voltage is reduced and the transient voltage–current curve is hysteretic. The main factor affecting the transition to soft sparks during PEO of aluminum alloys is the cathodic current. Cheng et al. [16] found that the transition to soft sparks is promoted by bipolar current conditions and an anodic-to-cathodic current ratio (Rpn) less than 1, preferably in the range of 0.8 to 1. The Rpn values of aluminum alloys were found to be in the range of 0.8 to 0.1. The smaller the value of the Rpn, the shorter the transition to soft sparking, but too low an Rpn value leads to premature arc quenching of the plasma. Melhem et al. [6] reported a similar behavior and found that the 2024 aluminum alloy in Na_2_SiO_3_ and KOH solution will transition to soft sparking when Rpb = 0.89. Martin et al., on the other hand, found that the transition to soft sparking in the PEO process of aluminum alloys is related to the current density and frequency, and the higher the frequency, the earlier the transition. In addition, it has recently been reported that the addition of organic additives to the PEO process of aluminum alloys promotes the transition to the soft spark discharge state. Hussain et al. [17] reported that when citric acid and oxalic acid were added as organic additives to alkaline borate electrolytes, the localized strong plasma discharges during the PEO process of aluminum alloys were significantly suppressed. Kamil et al. [18] concluded that the transition to the soft spark state during PEO of aluminum alloys in silicate electrolytes containing organic additives was significantly suppressed. In silicate electrolytes with organic additives, aluminum alloys will form an EDL layer on the surface of the substrate during the PEO process, and the EDL layer can provide a shielding effect by depleting the energy of the localized strong plasma discharges, thus promoting the transition to soft spark discharges. Although there are many reports on the soft spark transition during PEO of aluminum alloys, the growth behavior of coatings under soft spark discharge remains to be refined.

The aim of this paper is to investigate the growth behavior of coatings under soft spark discharge. By comparing the microscopic morphology, phase composition and elemental distribution of the coatings under different PEO treatment times, the film formation mechanism of the coatings under different discharge stages is proposed.

## 2. Experimental Procedures

### 2.1. Sample Preparation

The specimen used in this experiment was a cylindrical 6061 aluminum alloy of Φ35 × 3 mm. Its chemical composition as determined by EDS was as follows (wt%): 0.88 Mg, 0.50 Si, 0.34 Fe, 2.75 O, 0.258 Cu, 0.09 Zn, 0.12 Cr, 0.04 Ti, 0.14 Mn and balanced Al. Prior to the experiments, the surfaces of the specimens were sanded to an average roughness of about 0.1 μm using silicon carbide sandpaper, and then the specimens were degreased in an ultrasonic cleaner by immersing them in a container with acetone and rinsed repeatedly with deionized water before drying.

### 2.2. Plasma Electrolytic Oxidation

The PEO process was carried out in an electrolyte of anhydrous sodium silicate (15 g/L), sodium hexametaphosphate (5 g/L) and sodium hydroxide (3 g/L). The temperature was controlled at about 15 ± 2 °C using a cooling circulation system. The specimen (as anode) and a stainless-steel coil (as cathode) were connected to a pulsed power supply with electrical parameters set as shown in Table 1.

### 2.3. Methods for the Study of Coating Properties

The thickness was measured using an eddy current thickness gauge (Helmut Fischer GMBH, Berlin, Germany) at five randomly selected points on each side of the specimen (accuracy: 0.1 μm), and the average value indicated the thickness of the coating. The surface roughness of the plasma electrolytic oxidation coatings was measured using a roughness tester (TR-210, Shanghai, China), and the crystalline composition of the PEO coatings was examined by a D/Max-2400 X-ray diffractometer (XRD; Cu Kα radiation) with a sweep angle of 3°, a scanning range of 10–90°, a step size of 0.02° and a scanning speed of 8°/min. The microscopic morphology of the PEO coatings was characterized using a scanning electron microscope (SEM; JSM-IT500, Akishima, Japan) equipped with an X-ray energy spectrometer (EDS; X-ACT). The emission spectroscopies from the discharge sparks during the PEF process were studied by an optical emission spectrometer (OES; Ideaoptics PG2000-Pro, Shanghai, China).

## 3. Results

### 3.1. Voltage-versus-Time Curves and Spark Morphology Evolution during the PEO Process

**Figure 1:**
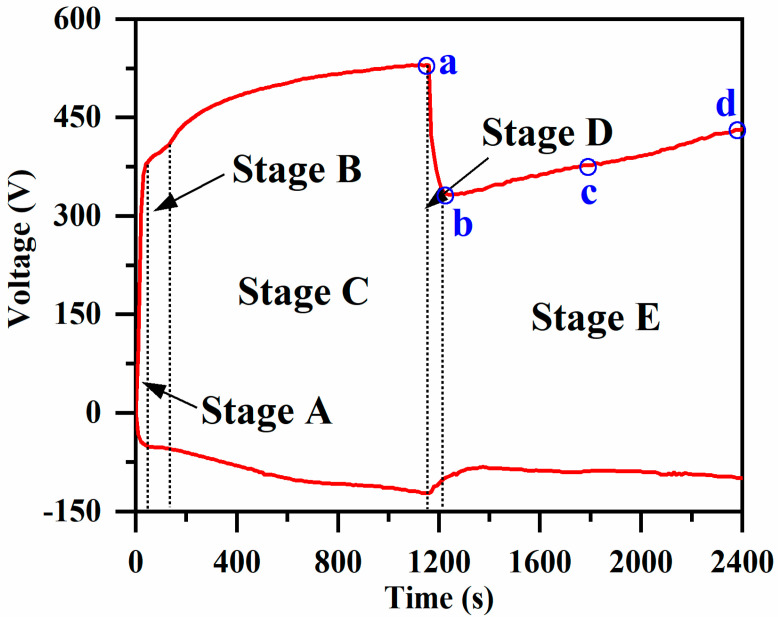
Trend of surface voltage of a sample over time under soft spark discharge for different time periods.

Figure 1 shows the time–voltage diagram of the PEO treatment process of aluminum alloys under bipolar conditions in silicate and phosphate composite systems, which can be roughly divided into five stages.

**Figure 2:**
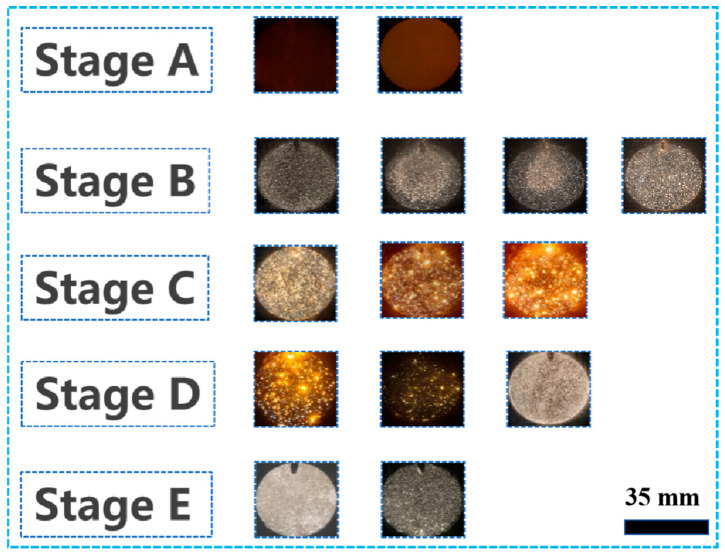
Surface plasma discharge morphology of samples at different stages.

Stage A, the first stage, is the anodic oxidation stage, which does not produce spark discharge and is often referred to as the non-spark discharge stage (Figure 2). The process has a relatively short duration of about 80 s, which serves to generate a kind of amorphous insulating film on the surface of the substrate to provide the necessary conditions for the subsequent dielectric breakdown [19]. In Stage A, the forward voltage increases linearly and rapidly with a large slope, a large number of bubbles are observed on the surface of the sample and the thickness of the oxide film increases, which brings about a gradual decrease in the electrical conductivity and an increase in the insulating property.

The second stage (Stage B) is the spark discharge stage, which can be clearly observed on the surface of a sample as dense and small white sparks, as shown in Figure 2, which, interestingly, shrink from the edge to the center and then are uniformly distributed over the entire surface of the sample. The reason for this phenomenon may be that, in the discharge area, the electric field intensity is not uniformly distributed and there exists a certain gradient because the electric field intensity is proportional to the voltage gradient, thus leading to a larger electric field intensity at the edge of the discharge area and a smaller electric field intensity in the middle part. This electric field distribution will result in the spark discharge starting from the edge and then gradually spreading to the center. According to the literature [20], some scholars believe that the cause of this phenomenon is related to gas density. In the process of spark discharge, the gas around a spark will be heated and expanded, forming a local density gradient of the gas, and, due to the high density of the gas at the edge of the spark discharge region and the low density of the gas in the middle part of the region, it is easier for a spark to form at the edge and propagate to the middle. This phase lasts about 80 s to 180 s, and then the white spark changes to an orange spark, which also signals the arrival of the third phase.

The third stage (Stage C), also known as the micro-arc discharge stage, can be observed after 185 s with the growth of the forward voltage with the processing time and increases steadily, but this stage produces a strong spark discharge that will produce a number of adverse effects on the PEO coating. After about 1160 s, the arc light will change from a strong orange spark to a soft orange spark, which means that the micro-arc discharge starts to change to a soft spark discharge, and the forward voltage reaches a peak of about 505 V at this time.

The fourth stage (Stage D) is the transition to soft spark discharge, which exists for about 40 s. This stage exists for a relatively short period of time and is therefore also called the transition stage. In Stage D, the orange sparks on the surface of the sample become fainter and darker and then change to small snow-white sparks, which are often called soft spark discharges. Along with this, the process is accompanied by a decrease in acoustic emission, consistent with the soft spark discharge phenomenon documented in the literature [21]. The voltage decreases from 505 V to about 302 V—a voltage drop of 40.19%. He et al. [22] found that the drop increases with increasing current density.

The fifth stage (Stage E) is the soft spark discharge stage, in which small snow-white sparks are always uniformly distributed on the surface of the sample, and the voltage will again increase with the treatment time.

**Figure 3:**
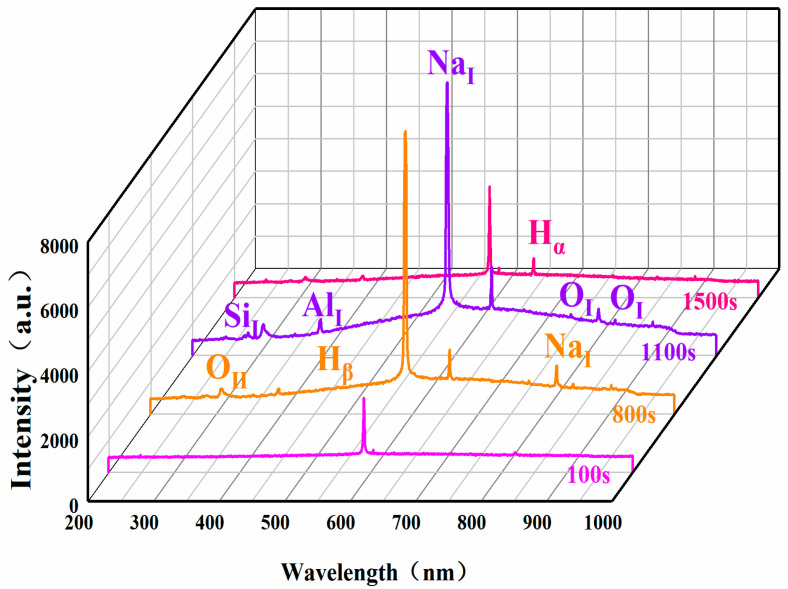
OES spectra of different treatment times in the PEO process.

In order to prove that the transition to soft spark discharge was successful, OES spectra were measured for the PEO process at different times, and the results are shown in Figure 3. At 100 s, only a NaI peak (589.5 nm) appeared; at 800 s, on the basis of the original peaks, an OII peak (304.2 nm), an AlI peak (396.1 nm) and a Hβ peak (486.1 nm) appeared; at 1100 s, there appeared a SiI peak (288.1 nm), and the peaks reached their highest values; and at 1500 s, the transition to soft spark discharge was completed and the peaks showed an obvious plunge, with the intensity of the optical emission decreasing by about 90%.

### 3.2. Effect of Soft Spark Discharge on Phase Composition

**Figure 4:**
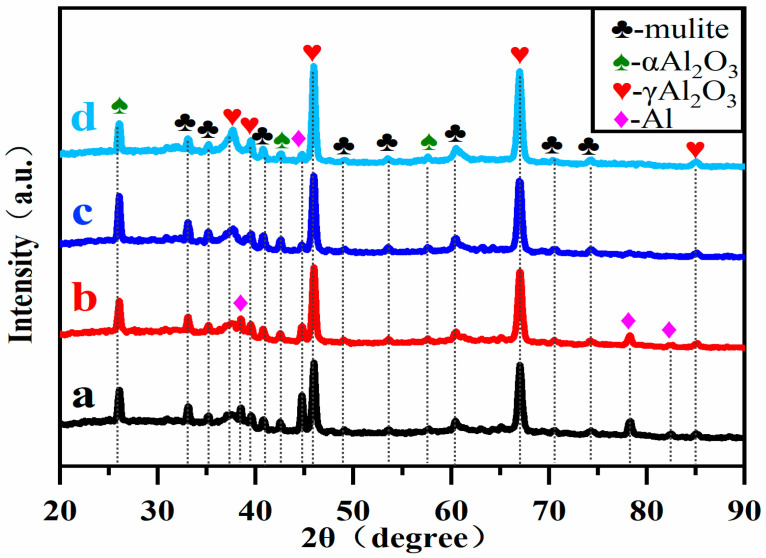
XRD patterns of PEO ceramic layers under soft spark discharge treatment at different times.

Figure 4 shows the XRD patterns of PEO ceramic layers under soft spark discharge for different time periods. The diffraction peaks of Al, α-Al_2_O_3_, γ-Al_2_O_3_ and mullite can be seen in the figure. From a to d, it can be observed that the diffraction peak intensities of α-Al_2_O_3_ and mullite increase and then decrease with the treatment time, and the diffraction peak intensity of Al decreases while the diffraction peak intensity of γ-Al_2_O_3_ increases.

As is known from previous studies [23,24], the relative phase contents of α-Al_2_O_3_ and γ-Al_2_O_3_ in the PEO ceramic layer can be obtained by the ratio of the maximum diffraction peak intensity of α-Al_2_O_3_ to that of γ-Al_2_O_3_, I_α_/I_γ_, and the results are shown in Table 2. From the beginning of the transition to soft spark discharge (a) to the completion of the transition to soft spark discharge (b), the relative phase content of Al_2_O_3_ increases while I_α_/I_γ_ decreases. The decrease in the I_α_/I_γ_ value indicates that there is a transition from B-type strong discharge to D-type and E-type discharge, which leads to a reduction in the generated heat and then to the difficulty in the transition of γ-Al_2_O_3_ to α-A_l2_O_3_, which causes a decrease in the I_α_/I_γ_ value. It has been documented [25] that the sintering of mullite occurs at 1923 K. It is precisely the decrease in the heat generated during this transformation process that leads to a decrease in the relative phase content of mullite. In the period from the completion of the transformation (b) to the soft spark discharge treatment for 20 min (d), the mullite and I_α_/I_γ_ values increase and then decrease, implying that a short period of soft spark discharge treatment leads to an increase in the temperature inside the ceramic layer. In addition, the decrease in the relative phase content of Al is due to the inward growth of the film layer under soft spark discharge.

### 3.3. Effect of Soft Spark Discharge on the Microscopic Morphology of Ceramic Layers

#### 3.3.1. Effect of Soft Spark Discharge on the Surface Morphology of Ceramic Layers

**Figure 5:**
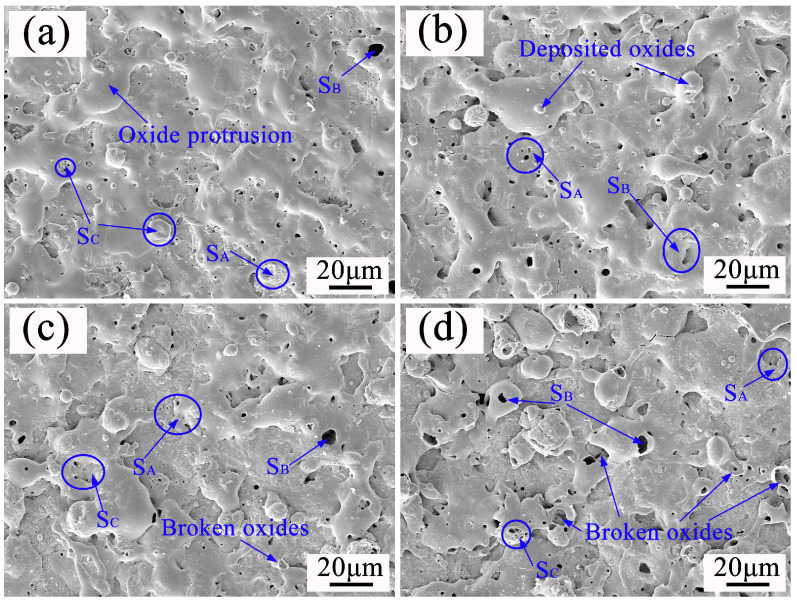
Surface morphology of PEO ceramic layer under soft spark discharge treatment at different times: (**a**) 1160 s; (**b**) 1200 s; (**c**) 1800 s; (**d**) 2400 s.

**Figure 6:**
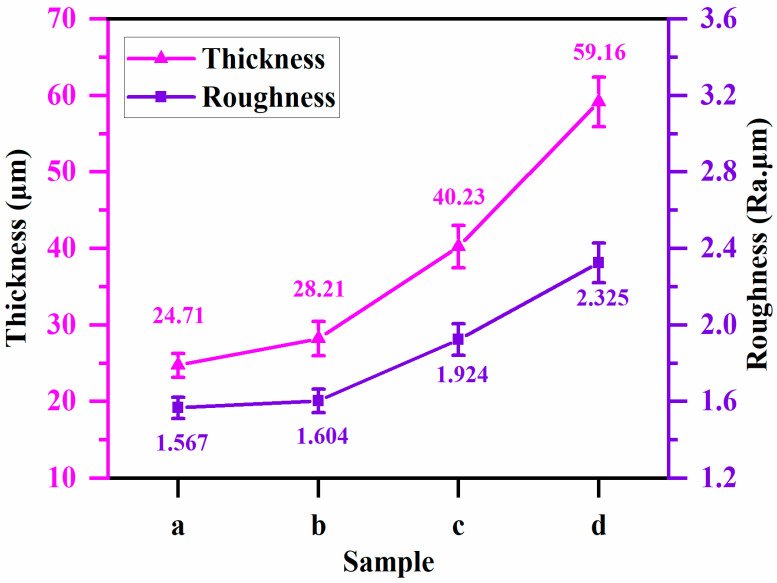
Thickness and roughness-versus-time curves for PEO ceramic layers under soft spark discharge treatment for different times.

**Figure 7:**
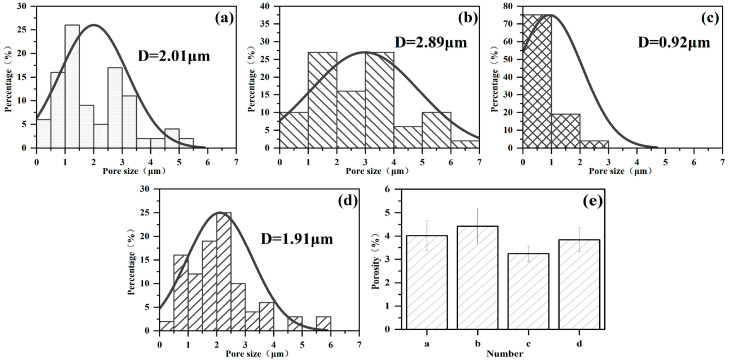
Pore size distribution and porosity on the surface of PEO ceramic layers under soft spark discharge treatment at different times (**e**): (**a**) Just starting to shift to soft spark discharge; (**b**) Complete transformation to soft spark discharge transformation; (**c**) Soft spark discharge transformation treatment for 10 min; (**d**) Soft spark discharge transformation treatment for 20 min.

Figure 5 shows the surface morphology of a PEO ceramic layer under soft spark discharge treatment for different times. Figure 5a shows the ceramic layer prepared at the beginning of the soft spark transition (1160 s), with a protruding porous oxide on the surface, i.e., the SA-type discharge morphology. The SA-type discharge pattern is a hollow structure with bubble holes of about 2 μm in diameter in the upper part, and the SB-type discharge pattern is a discharge channel left by the strong B-type discharge. In addition, a bowl-shaped crater (SC) was found on the surface of the ceramic layer, which is different from the conventional volcanic accumulation pattern, with a large number of pores of about 1 μm in diameter at the bottom that have sharp edges but are smooth inside. When completely transformed to soft spark discharge (1200 s), it [25] can be seen that the number of protruding oxides on the surface of the ceramic layer increases and that amorphous oxide particles begin to form, as has been pointed out in the literature, which contain a large amount of Si in the glassy state; the substance often appears in the form of a deposition, which leads to an increase in the roughness of the ceramic layer, as shown in Figure 6. It can be seen that when the soft spark discharge treatment was applied for 10 min (1600 s), as shown in Figure 4, Figure 5, Figure 6 and Figure 7, the porosity on the surface of the ceramic layer was reduced and the average pore size became smaller, but the number of protruding oxides and deposited particles increased. The decrease in porosity may be due to the fact that some of the oxides in the ceramic layer melted to fill the original pores during the soft spark discharge process. With a longer treatment time (2400 s), the pore size on the surface of the ceramic layer increased again, and the porosity increased but remained lower than the pore size and porosity at 1200 s. However, the crushed oxides became more and more obvious and the particles were larger, which may be attributed to the following processes: (1) the soft spark discharge process causes localized melt flow on the metal surface, and this flow leads to changes in the structure of the surface particles, which lead to the particles becoming larger; (2) as the soft spark discharge continues, the previously formed pores gradually increase in the subsequent treatment, as shown in Figure 5 which makes the surface particles increase in size; (3) the soft spark discharge process undergoes intense gas evolution, and the resulting bubbles expand at high temperatures, affecting the surrounding particles.

Overall, in terms of the surface morphology of the ceramic layer alone, the short-time soft spark discharge treatment (c) repaired the pore size and pores of the film layer, while the long-time soft spark discharge treatment (d) increased the pore size again, from 0.92 μm to 1.91 μm, as can be seen in Figure 7c,d. Combined with the analysis, and as can be seen in Figure 5c,d, the increase in the number of surface particles also led to an increase in the surface roughness from 1.924 μm to 2.325 μm. In addition, it can be seen in Figure 6 that the film formation rate under the long-time soft spark discharge treatment was larger than that under the short-time soft spark discharge treatment. The reason for this is that, under the long-time soft spark discharge treatment, the ceramic layer grew predominantly inward, which accelerated the rate of the Al substrate’s participation in the oxidation reaction, resulting in a faster film formation rate.

#### 3.3.2. Effect of Soft Spark Discharge on the Cross-Sectional Morphology of Ceramic Layers

**Figure 8:**
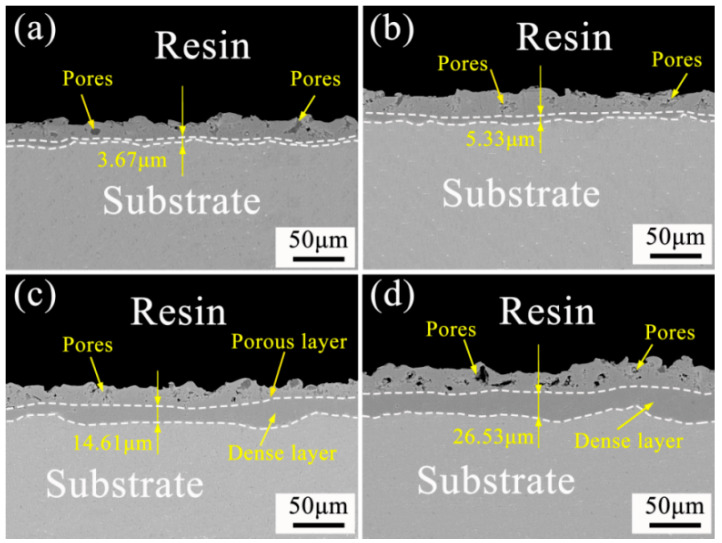
Cross-sectional morphology of PEO ceramic layer under soft spark discharge treatment at different times: (**a**) 1160 s; (**b**) 1200 s; (**c**) 1800 s; (**d**) 2400 s.

Figure 8 shows the cross-sectional morphology of a PEO ceramic layer under soft spark discharge treatment for different times. The ceramic layer can be roughly divided into two layers, i.e., the outer layer, also called the loose layer, and the dense layer. With the continuation of the treatment time, the dense inner and outer layers gradually thicken, and the interface between the substrate and the ceramic layer shows a wavy shape. As can be seen from Table 3, the thickness of the ceramic layer of sample a is about 24.71 μm, the thickness of the dense layer is about 3.76 μm, and the thickness of the outer layer is about 20.95 μm; the thickness of the ceramic layer of sample b is about 28.21 μm, the thickness of the dense layer is about 5.33 μm, and the thickness of the outer layer is about 22.88 μm; the thickness of the ceramic layer of sample c is about 40.23 μm, the thickness of the dense layer is about 14.61 μm, the thickness of the dense layer was about 25.62 μm, and the thickness of the outer layer was about 25.62 μm; the thickness of the ceramic layer of specimen d was about 59.16 μm, the thickness of the dense layer was about 26.53 μm, and the thickness of the outer layer was about 32.63 μm. The film formation rate is about 0.014 μm/s before the transition to soft spark discharge and is about 0.014 μm/s during the transition to soft spark discharge. The film formation rate during the transition to soft spark discharge is about 0.088 μm/s, and the film formation rate during the soft spark discharge treatment is about 0.026 μm/s. The film formation rate of the PEO ceramic layer under soft spark discharge is much higher than that of the conventional PEO ceramic layer, which is in agreement with what has been documented in the literature [26,27,28,29]. It is noteworthy that the film formation rate during the transition to soft spark discharge is much higher than that during soft spark discharge—a phenomenon that has not been reported.

In addition, from the moment of transition to soft spark discharge, the dense part of the ceramic layer begins to thicken due to a change in the type of discharge from the previous strong B-type to E-type and D-type discharge. Under the influence of the low breakdown principle, the soft spark acts directly on the defects, resulting in a high temperature inside, which melts the generated oxide ceramic layer, and in the process of solidification, the organization is restructured, repairing micropores and cracks to increase the thickness of the dense layer. Analyzed in conjunction with Figure 4, this high temperature will indirectly lead to the transformation of the Al_2_O_3_ crystalline type, and the content of α-Al_2_O_3_ and mullite increases in the process from b to c. However, with the prolongation of the treatment time, the heat generated by the D and E discharges is much lower than that of the B-type strong discharge, which leads to the difficulty of the transformation of the crystalline form, resulting in the decrease in the content of α-Al_2_O_3_ and mullite in the process from c to d.

### 3.4. Direction of Ceramic Layer Growth before and after Soft Spark Discharge

**Figure 9:**
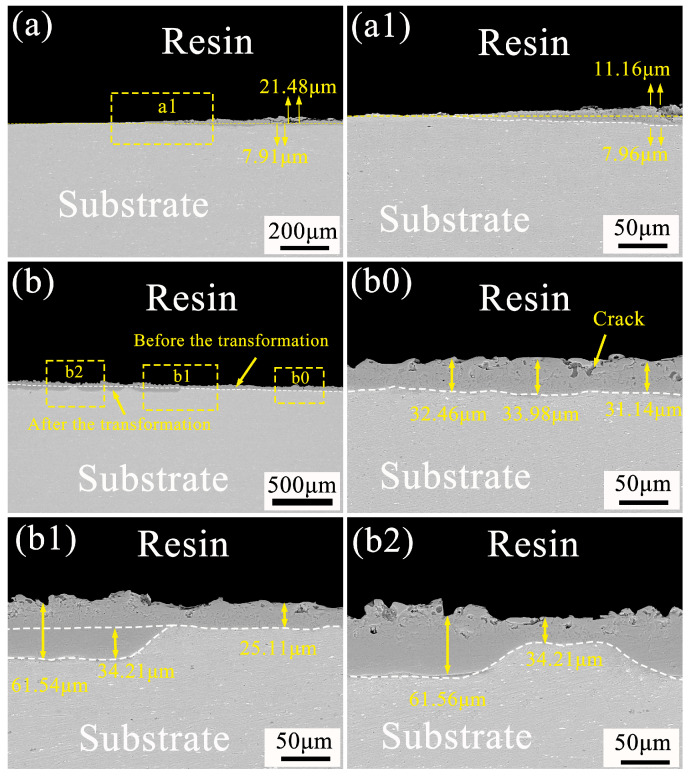
Microscopic cross-sectional morphology of PEO ceramic layer before and after soft spark discharge: (**a**) before soft spark discharge; (**b**) after soft spark discharge; (**a1**,**b0**,**b1**,**b2**) are localized area enlargements.

A homemade fixture was used to investigate the growth direction of the ceramic layer before and after the soft spark discharge in the bipolar PEO process. As shown in Figure 9, it is mainly divided into the growth characteristics of the ceramic layer before the soft spark discharge transition (Figure 9a and Figure 9(a1)) and the growth characteristics of the ceramic layer after the soft spark discharge transition (Figure 9b, Figure 9(b0), Figure 9(b1) and Figure 9(b2)). By comparison, it can be clearly observed that the growth patterns of PEO ceramic layers before and after soft spark discharge are very different. From Figure 9a, it can be seen that the growth mode before soft spark discharge is both inward and outward—the outward growth is 21.48 μm and the inward growth is 7.91 μm—and is mainly dominated by the outward growth. Figure 9(a1) shows the local enlargement, which shows that there are 11.16 μm of outward growth and 7.96 μm of outward growth under the influence of the fixture, and a large number of defects can be seen in the outward growth part. In Figure 9b, before and after the soft spark discharge growth mode, the b0 region for the soft spark discharge point before the region can be seen in the ceramic layer, the substrate interface line is approximately a straight line and the film thickness difference is small (Figure 9(b0)), while after soft spark discharge and the obvious changes in the ceramic layer, the substrate interface line is wavy and the film thickness difference is large (Figure 9(b2)); the thickest area is 61.56 μm, while the thinnest area is 34.21 μm, and the thickness of the ceramic layer before soft spark discharge is not much different, from which it can be inferred that the soft spark discharge is a localized discharge that only acts on a certain area. Figure 9(b1) shows the cross-section morphology near the fixture, which shows that the film layer basically rarely grows outward but mainly inward, and it grows to 34.21 μm. Combined with the analysis in Table 3, it was found that the film formation rate increased from 0.088 μm/s to about 0.026 μm/s when soft spark discharge was applied, which is more than twice the rate under the soft spark discharge treatment. When analyzed in conjunction with the XRD patterns shown in Figure 4, the reason for the outward thickening of the film layer during the soft spark discharge process may lie in the deposition of oxides, as a weak and wide bump appears in the region of 30~40°, which may be amorphous SiO_2_ or an amorphous phase material made of (Al_2_O_3_)-(SiO_2_) in combination with the electrolyte components, as described in the literature. When analyzed in conjunction with Table 2, it was found that the thickening of the inner layer was mainly due to the generation of Al_2_O_3_.

**Figure 10:**
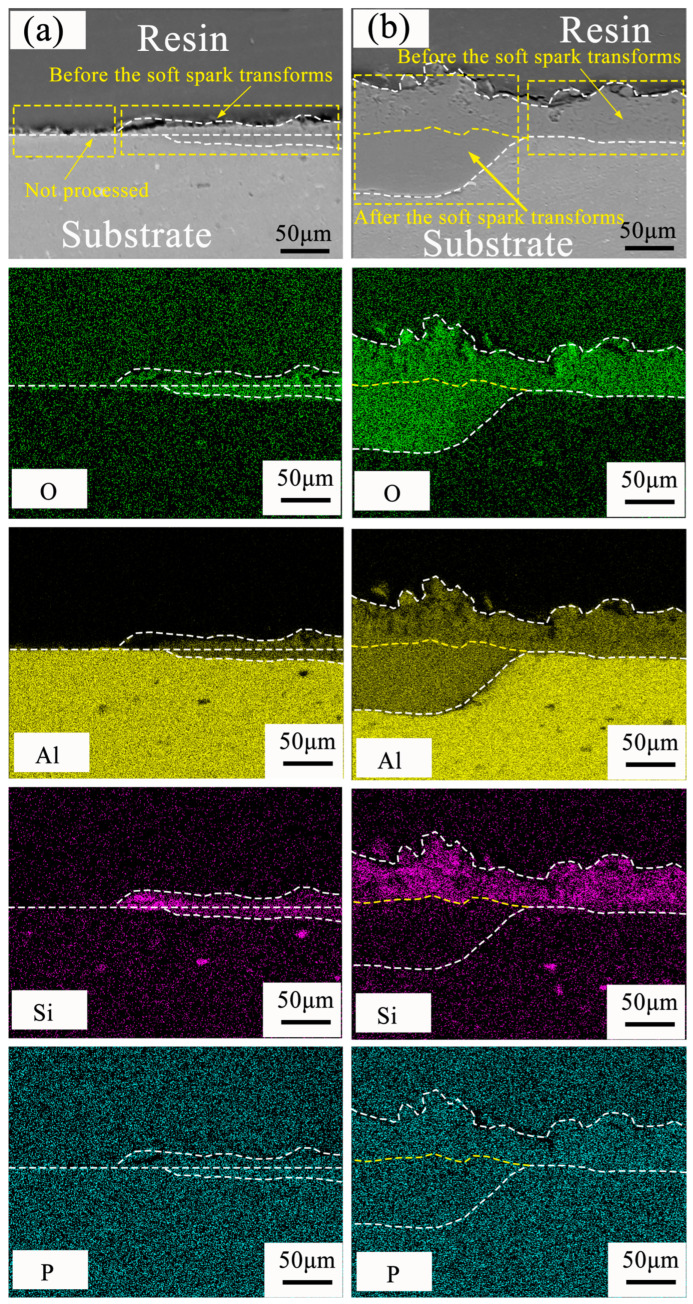
EDS elemental distribution of PEO ceramic layer in cross section before and after soft spark discharge: (**a**) Elemental distribution before soft spark discharge transformation; (**b**) Elemental distribution under soft spark discharge.

In order to further understand the film formation mechanism of the ceramic layer under soft spark discharge, EDS elemental analyses of (a1) and (b1) in Figure 9 were performed, and the results are shown in Figure 10. P elements could be found everywhere in the ceramic layer, which originated from P_6_O_18_^6−^ in the electrolyte, though we will not expand on this explanation here. O and Al elements were distributed throughout the ceramic layer, and, in Figure 10a, it can be clearly seen that O was enriched in the inward-growing ceramic layer and that Si was enriched in the outward-growing ceramic layer. In Figure 10b, it can be seen that, after the soft spark discharge, O and Al were enriched in the dense inner layer and the thickness of the outer ceramic layer seems to have decreased. Si was present in the outer layer and increased with the thickness of the outer layer, whereas there was almost no participation in the reaction in the inner layer. This phenomenon was first discovered in the study of aluminum alloys regarding soft spark discharges.

## 4. Discussion

Based on the results obtained from the above experiments, the following reasoning was made regarding the film formation mechanism of PEO ceramic layers under soft spark discharge.

As shown in Figure 1, the growth of voltage in the PEO process will go through five stages sequentially, including anodic oxidation, spark discharge, micro-arc discharge, soft spark discharge transition and soft spark discharge. Combining Figure 2 with the literature [20,26], in the anodic oxidation stage, the substrate Al is first electrolyzed to Al^3+^ under the action of a high electric field (Equation (1)), and Al^3+^ and oxygen-containing anions in the electrolyte (P_6_O_18_^6−^, SiO_3_^2−^, OH^−^ and O^2−^) are moved in the oxide under the action of a high electric field (E). Among them, Al^3+^ migrate toward the solid (membrane)–liquid interface, and anions such as P_6_O_18_^6−^, SiO_3_^2−^, OH^−^ and O^2−^ migrate toward the liquid–solid (membrane) interface. Assuming that ideal conditions obtain, it can be inferred from Equation (2) that OH^−^ are the first to arrive at the solid–liquid interface to participate in the reaction (Equation (3)) to generate Al(OH)_3_ and AlOOH (Equation (4)), and the substance is amorphous, so the anodic oxidation stage mainly generates an amorphous layer, with the main constituent being water-containing alumina [27].

(1) $$Al-{3e}^{-}\to {Al}^{3+}$$

(2) $$Eq={mv}^{2}$$

(3) $${Al}^{3+}+{OH}^{-}\to {Al(OH)}_{3}$$

(4) $${Al(OH)}_{3}-H_{2}O\to AlOOH$$

**Figure 11:**
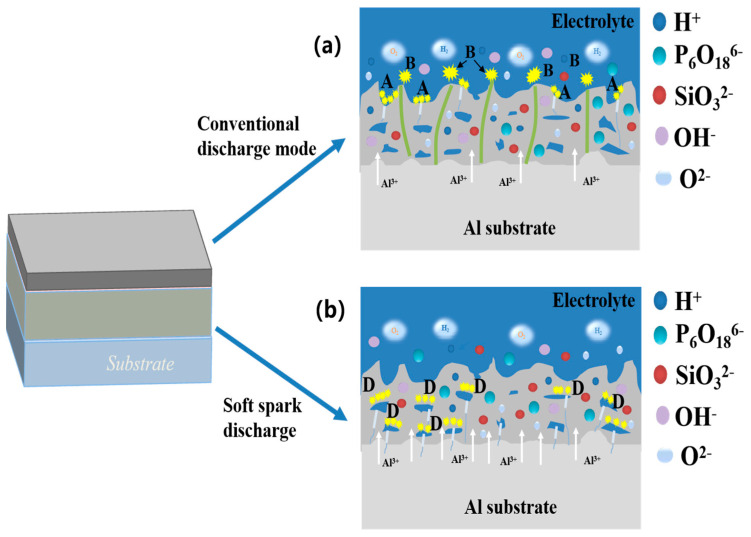
Two discharge models for the PEO process: (**a**) before soft spark discharge; (**b**) after soft spark discharge.

If the spark discharge stage (Figure 2, Stage B) and the micro-arc discharge stage (Figure 2, Stage C) are similar, given sample surface plasma spark discharge, the ceramic layer morphology of the disk and the solid–liquid interface oxidation reaction, the electrolyte contact with the ceramic layer interface begins to precipitate a large number of gases (Equations (6)–(8)). It has been shown in the literature that, as shown in Figure 11, type A (gas–liquid interface) discharge, type B (solid–liquid interface) discharge and type C (solid–solid interface) discharge mainly occur at this stage. Type A (gas–liquid interface) discharge prompts solute components in the electrolyte to take part in the film formation reaction, and the formation of the surface topography is shown in Figure 5 SA. Not only is type B (solid–liquid interface) discharge strong, the temperature can be as high as 4000–5000 K [3]. On the one hand, it can promote the dissolution and oxidation reaction of the substrate; on the other hand, the molten Al and Al_2_O_3_ can flow out in the solid–liquid direction by virtue of the discharge channel, which is also the reason for the outward diffusion of Al elements in Figure 10a. C-type (solid–solid interface) discharge, on the other hand, is mainly located between the barrier layer and the porous outer layer. This stage mainly involves the following reactions:

(5) $${2OH}^{-}-{2e}^{-}\to H_{2}O+[O]$$

(6) $$+[O]\to O_{2}↑$$

(7) $$H^{+}+{2e}^{-}\to H_{2}↑$$

(8) $$2H_{2}O+{2e}^{-}\to H_{2}↑+{OH}^{-}$$

(9) $${Al}^{3+}+O^{2-}\to {Al}_{2}O_{3}$$

In addition, from the detection of mullite in the XRD patterns shown in Figure 4, it is hypothesized that the following reactions may also occur:

(10) $$SiO_{3}^{2-}-{2e}^{-}\to {SiO}_{2}+[O]$$

(11) $$SiO_{3}^{2-}\to {SiO}_{2}+1/2O_{2}↑+{4e}^{-}$$

(12) $$n{SiO}_{2}+{Al}_{2}O_{3}\to {Al}_{2}O_{3}\cdot n{SiO}_{2}$$

SiO_2_ (nodular in Figure 5) is derived from sodium silicate in the electrolyte, and SiO_3_^2−^ decomposes around 2000 K to produce SiO_2_, which reacts with Al_2_O_3_ ejected along the discharge channel to form mullite. Analyzed in conjunction with Figure 10a, SiO_3_^2−^ can also enter the substrate–ceramic layer interface along the discharge channel to participate in the reaction for film formation. However, in Figure 10a, the Si content in the outer layer is significantly higher than that in the inner layer, which may be attributed to the fact that SiO_3_^2−^ ions migrate at a slow rate and have a large size and can only enter the inner layer through the large discharge channels (A, B and C). Some scholars have studied this and found that near the mouth of the B-type discharge channel in the inner layer, a large amount of silicon is enriched. Al_2_O_3_ originates from the reaction between O^2−^ migrating to the solid–liquid interface and Al^3+^ located at the interface of the substrate–ceramic layer on the one hand, and, on the other hand, O^2−^ may also react directly with molten Al in the discharge channel to produce Al_2_O_3_. In addition to this, Al_2_O_3_ also originates from the decomposition of Al(OH)_3_ and AlOOH, which is accompanied by the transformation of the aqueous amorphous alumina phase to the crystalline alumina phase via the following reaction:

(13) $${Al(OH)}_{3}\overset{1123-1473\;K}{\to }{Al}_{2}O_{3}+H_{2}O$$

(14) $$2AlOOH\overset{723-1073K}{\to }\gamma {-Al}_{2}O_{3}+H_{2}O$$

Al_2_O_3_ has an α-Al_2_O_3_ phase in addition to the γ-Al_2_O_3_ phase, and it has been reported [28] that γ-Al_2_O_3_ transforms to α-Al_2_O_3_ when the temperature is in the range of 1323–1473 K, as shown in Equation (15).

(15) $$\gamma {-Al}_{2}O_{3}\overset{1323-1473K}{\to }\alpha {-Al}_{2}O_{3}+H_{2}O$$

In the soft spark transition stage (Figure 1, Stage D), micro-arc discharges coexisted with soft spark discharges, and there were D-type (at the substrate–inner layer interface) and E-type (at the outer layer–electrolyte interface) discharges in addition to the A-, B- and C-type discharges. This stage leads to a reduction in the number of cracks and defects in the ceramic layer due to the appearance of D-type and E-type discharges, as shown in Figure 8b. When analyzed in conjunction with Figure 6, this stage lasts for a short time of about 40 s, but the film formation rate is as high as 0.088 μm/s, which is about four times that of the film formation rate under the soft spark discharge treatment, and, in addition to the inward growth of the ceramic layer brought about by the D-type discharges, the deposition rate of the outer layer of the ceramic layer is also increased under the influence of the change in the type of the discharges.

The soft spark discharge stage (Figure 11b), which is analyzed in conjunction with the OES spectra in Figure 3, is dominated by D-type discharges and supplemented by E-type discharges, with almost no B-type discharges. The E-type discharges do not produce penetration holes, although they also carry disc-like structures on the surface of the ceramic layer. The film layer produced in this stage is not uniform, as can be seen in Figure 9(b2); the thickest area is 61.56 μm, while the thinnest area is 34.21 μm. However, it can be seen that the number of pores and defects in the whole ceramic layer is significantly lower than in the ceramic layer before soft spark discharge, but the thinnest area still has more through-holes and cracks, and in the thinnest area the soft spark discharge stage does not seem to change, meaning that soft spark discharge is the main reason for the increase in the number of pores and defects, though the thinness of the ceramic layer is the most important factor. This means that the soft spark discharge is localized. Combined with Figure 10b, in the soft spark discharge treatment process, with the disappearance of B-type discharge, the ceramic layer’s inward growth part almost does not contain Si elements, meaning that only through the B-type discharge channel can the substrate–ceramic layer interface participate in the film formation reaction. However, it can be seen from Table 2 that the mullite content in the ceramic layer still increases after a short time of soft spark discharge treatment, implying that the temperature of the ceramic layer increases for that short time. The reason for this is the presence of a Si-bearing inorganic layer in the outer layer of the coating [29], and the weak and wide bumps in the region from 30° to 40° in Figure 4 are indicative of the presence of an inorganic layer in the ceramic layer. The Si-bearing inorganic layer provides high thermal barrier properties, which leads to an increase in the temperature within the ceramic layer, and the nucleation of mullite occurs at 1200 K [30,31], which in turn leads to an increase in the size of the particles on the surface of the ceramic layer, as shown in Figure 5c,d. Analyzed in conjunction with Table 2, this temperature increase also indirectly leads to the increase in the α-Al_2_O_3_-phase content in the ceramic layer. And, under the long-time soft spark discharge treatment, the intensity of D-type discharge is significantly lower than that of B-type discharge, which generates less heat, and the accelerated cooling circulation system leads to a decrease in the internal temperature in the ceramic layer, which in turn leads to newly generated γ-Al_2_O_3_ that cannot be transformed to α-Al_2_O_3_ [32], which leads to a decrease in the content of α-Al_2_O_3_ and mullite phases.

**Figure 12:**
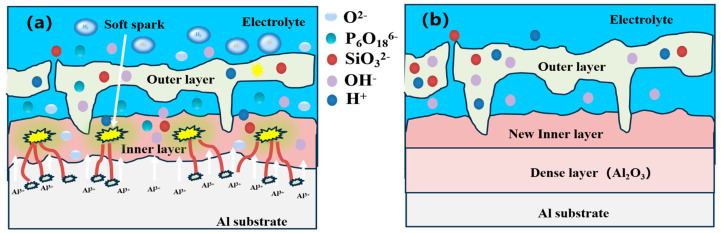
Modeling of ceramic layer growth before and after soft spark discharge during PEO: (**a**) before soft spark discharge; (**b**) after soft spark discharge.

In summary, Figure 12a shows the growth model during the soft spark discharge transition, and it can be seen that this process of soft spark discharge is mainly an internal D-type discharge, and P_6_O_18_^6−^, OH^−^ and O^2−^ diffuse or migrate into the ceramic layer to participate in the reaction through the pores and the discharge channel, whereas SiO_3_^2−^ is unable to enter the ceramic layer because of its large size and low migration rate and participates in the film formation only on the surface of the outer layer through deposition. The soft spark discharge process is mainly one of inward growth; Al^3+^ and OH^−^ and O^2−^ plasma reaction growth of Al_2_O_3_, due to the D-type discharge being more moderate, will not produce pores and other defects, resulting in the existence of a dense layer containing Al_2_O_3_ inside the ceramic layer; in addition, the direct generation of D-type discharge will occur in the original pores near the melting of the tissue and fill in the pores to form a new layer, and the new inner layer and the dense layer will together form a barrier layer, and the result is shown in Figure 12b.

## 5. Conclusions

1. Relative to the substrate, the growth direction of the PEO ceramic layer before the soft spark discharge transition is dominated by outward growth. The growth direction of the PEO ceramic layer after the soft spark discharge transition is dominated by inward growth relative to that before the soft spark discharge transition.
2. After soft spark discharge, the Si element is distributed in the outer layer, which is due to the fact that SiO_3_^2−^, on account of its large size, cannot enter the interface between the substrate and the inner layer of the ceramic layer to participate in the film formation through the D-type discharge channel.
3. The relative phase content of α-Al_2_O_3_ in the coating increased by 30.2% from 0.487 to 0.634 after 10 min of trans-spark discharge compared to that before the soft spark discharge transformation, while the relative phase content of α-Al_2_O_3_ in the coating decreased by 55.6% from 0.487 to 0.313 after 20 min of trans-spark discharge compared to that before the soft spark discharge transformation.
4. The film formation rate was 0.014 μm/s before the soft spark discharge transition and 0.026 μm/s after the soft spark discharge transition, and, comparing the two, the film formation rate was improved by 85.14% under soft spark discharge.

## Figures and Tables

**Table 1:** Names of different samples and electrical parameters used in their preparation.

Specimen Number	a	b	c	d
Frequency (Hz)	500			
Duty cycle (%)	+20%, −20%			
Current density (A/dm^2^)	+6, −7.5			
Processing time (s)	1160	1200	1800	2400

**Table 2:** Relative phase content of PEO ceramic layers under soft spark discharge treatments of different duration.

Specimen Number	Al_2_O_3_ (wt%)	Mullite (wt%)	Al (wt%)	I_α_/I_γ_
a	60.5	33.1	6.4	0.487
b	63.8	32.3	3.9	0.408
c	64.7	34.2	1.1	0.634
d	70.6	28.4	1.0	0.313

**Table 3:** Thickness variations of dense and outer layers of PEO ceramic layers under soft spark discharge treatments of different durations.

Specimen Number	Thickness of Dense Layer (μm)	Outer Layer Thickness (μm)	Coating Thickness (μm)
a	3.76	20.95	24.67
b	5.33	22.88	28.21
c	14.61	25.62	40.23
d	26.53	32.63	59.16

## Data Availability

Data are contained within the article.
